# Zinc and Paclobutrazol Mediated Regulation of Growth, Upregulating Antioxidant Aptitude and Plant Productivity of Pea Plants under Salinity

**DOI:** 10.3390/plants9091197

**Published:** 2020-09-14

**Authors:** Mahmoud R. Sofy, Khalid M. Elhindi, Saad Farouk, Majed A. Alotaibi

**Affiliations:** 1 Botany and Microbiology Department, Faculty of Science, Al-Azhar University, Cairo 11884, Egypt; 2 Department of Plant Production, College of Food and Agriculture Sciences, King Saud University, Riyadh 11451, Saudi Arabia; kelhindi@ksu.edu.sa (K.M.E.); malotaibia@ksu.edu.sa (M.A.A.); 3 Department of Vegetable and Floriculture, Faculty of Agriculture, Mansoura University, Mansoura 35516, Egypt; 4 Agricultural Botany Department, Faculty of Agriculture, Mansoura University, Mansoura 35516, Egypt; gadalla@mans.edu.eg

**Keywords:** antioxidant, field pea, paclobutrazol, salinity, yield, zinc

## Abstract

Soil salinity is the main obstacle to worldwide sustainable productivity and food security. Zinc sulfate (Zn) and paclobutrazol (PBZ) as a cost-effective agent, has multiple biochemical functions in plant productivity. Meanwhile, their synergistic effects on inducing salt tolerance are indecisive and not often reported. A pot experiment was done for evaluating the defensive function of Zn (100 mg/L) or PBZ (200 mg/L) on salt (0, 50, 100 mM NaCl) affected pea plant growth, photosynthetic pigment, ions, antioxidant capacity, and yield. Salinity stress significantly reduces all growth and yield attributes of pea plants relative to nonsalinized treatment. This reduction was accompanied by a decline in chlorophyll, nitrogen, phosphorus, and potassium (K^+^), the ratio between K^+^ and sodium (Na^+^), as well as reduced glutathione (GSH) and glutathione reductase (GR). Alternatively, salinity increased Na^+^, carotenoid (CAR), proline (PRO), ascorbic acid (AsA), superoxide dismutase (SOD), catalase (CAT), and ascorbate peroxidase (APX) over nonsalinized treatment. Foliar spraying with Zn and PBZ under normal condition increased plant growth, nitrogen, phosphorus, potassium, K^+^/Na^+^ ratio, CAR, PRO, AsA, GSH, APX, GR, and yield and its quality, meanwhile decreased Na^+^ over nonsprayed plants. Application of Zn and PBZ counteracted the harmful effects of salinity on pea plants, by upregulating the antioxidant system, ion homeostasis, and improving chlorophyll biosynthesis that induced plant growth and yield components. In conclusion, Zn plus PBZ application at 30 and 45 days from sowing offset the injuries of salinity on pea plant growth and yield by upregulating the antioxidant capacity and increasing photosynthetic pigments.

## 1. Introduction

Salinity is the foremost global ecological constraint to worldwide sustainable production and food security. Salt stress influences about 936 Mha of arable lands, causing yearly worldwide monetary losses of 27.5 billion USD [1,2]. Severe salinity induces different physio-biochemical abnormalities, including dual hyperosmotic effects, nutritional imbalance, specific ion toxicity, impaired gas exchange, disturbing water homeostasis, or a mixture of these factors, which reduce plant growth and yield [3,4,5]. Additionally, salinity stress manifests an overproduction of reactive oxygen species (ROS), like superoxide (O_2_^−^) and hydrogen peroxide (H_2_O_2_), which seriously disrupt normal metabolism and causes a drastic physio-biochemical and molecular dysfunction [6,7,8]. Plants under stress conditions activate self-protection strategies to mitigate oxidative injury and disposing of ROS molecules. These include exclusion and compartmentation of toxic ions, as well as overproduction of compatible solutes [3,7,9]. Together with nonenzymatic antioxidants (phenolic compounds, carotenoids ”CAR”, flavonoids “FLAV”, ascorbic acid “AsA”, and reduced glutathione “GSH”), plants nullify ROS by upregulating antioxidant enzyme activities that quench O_2_^−^ to H_2_O_2_, and finally into the water and molecular oxygen [7,10,11]. Moreover, the assimilation of antioxidant compounds work as a noticeable protection feature under the stressful condition that directly interacts with the detoxifying free radicals [4,12].

There are different ways to improve salinity tolerance in crops for ensuring food security, involving biotechnological methods and application of some activators [13]. Accordingly, evaluation of the possible functions of activators like micronutrient and plant growth substances offer an efficient explanation for the enhancement of plant resiliency to the unfavorable impacts of ever-changing ecological disorders [3,14,15].

Zinc (Zn) has numerous pivotal biochemical and molecular purposes in different plant species under normal or stressful conditions, including the upregulation of ROS scavenging strategies, the activation of several enzyme systems (about 300 enzymes), and improved nucleic acid biosynthesis [16,17,18,19]. Additionally, Zn is involved in indole acetic acid assimilation, cell development, and sexual reproduction [14]. It has been proposed that Zn supplementation is essential in plants’ protection strategies under salinity. Concerning this, Farouk and Al-Amri [3] documented that exogenous application of zinc on salt-affected canola plants significantly increased plant growth, photosynthetic pigments, ion concentration, and increased yield.

Phytohormones like triazole compounds (plant multi-stress protectants) play an imperative function in regulating various growth and behavioral processes under normal or stressful conditions [20]. Paclobutrazol (PBZ) is now extensively used in agriculture for regulating plant development and increasing crop yield under normal or stress conditions [14,21]. The most prominent and likely hypothesis on increasing plant production and stress tolerance induced by PBZ has been attributed to it sustaining the endogenous cytokinin concentration, maintaining water status, improving nutrient uptake and carbohydrate synthesis, improving chlorophyll biosynthesis, and promoting antioxidant capacity [20,21].

Field pea (*Pisum sativum* L.) is an attractive cool-season food legume consumed by humans in its green state as well as dry, in the form of pulse, due to its high content of protein (25–35%) and important amino acids [22]. In addition, the seeds are an excellent source of carbohydrates, minerals (calcium and iron), and vitamins (thiamin, tocopherols, niacin, and folic acid) [22]. Earlier reports have individually documented that zinc or PBZ, as cost-effective agents, have multiple biochemical functions in plant development under stressful conditions [14,23,24]. Conversely, their integrative application on inducing pea salt tolerance, to our knowledge, has not been documented and required more exploration. It is imperative to distinguish the biochemical approaches of pea plants in response to Zn and PBZ application under salinity. Therefore, the purpose of the current research was to assess the defensive interplaying roles of the combined Zn and PBZ application on pea growth and yield attributes, and its antioxidant capacity under salinity. It was assumed that application of Zn and PBZ successfully reduce the negative effects of salinity on pea productivity by reducing Na^+^, sustaining ion homeostasis, and upregulating the antioxidant system. The results described herein are anticipated to support the improvement of pea production on salt-affected soils.

## 2. Results

### 2.1. Plant Growth

**Figure 1:**
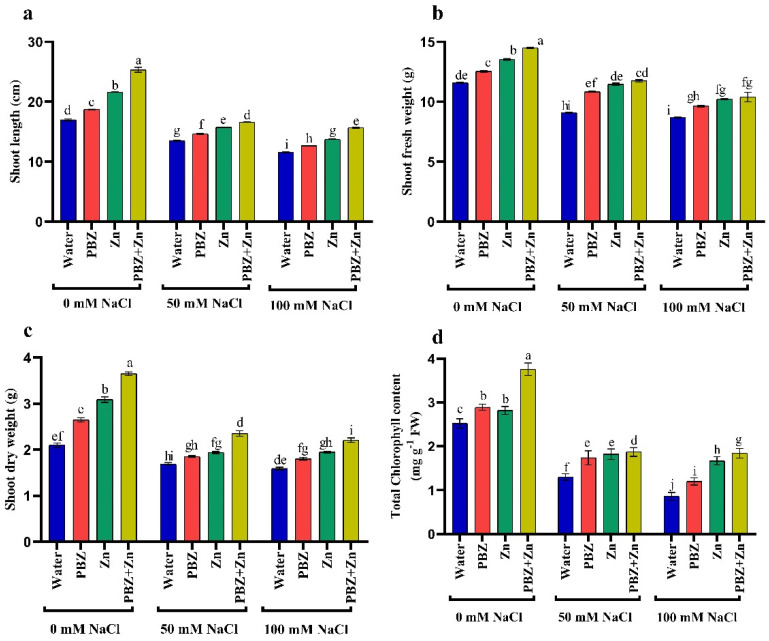
Effect of salinity level (0, 50, 100 mM NaCl) and applications of zinc (Zn), paclobutrazol (PBZ), and their interaction (Zn + PBZ) on (**a**–**c**) pea plant growth and (**d**) chlorophyll concentration at 50 days from sowing. Values (*n* = 5) in columns followed by the different letter (a, b, c, d, e, f, g, h, i, j) are significantly different, *p* < 0.05.

To realize the role of Zn or PBZ in field pea plant growth under salinity, we measured report shoot length (SL), shoot fresh (SFW) and dry (SDW) weights under normal or salinity conditions (Figure 1a–c). Field pea plants that were sprayed with Zn + PBZ showed noticeable differences in growth relative to the plants sprayed with either Zn or PBZ alone, and untreated control plants (Figure 1a–c). The highest values of SL (49%), SFW (25%), and SDW (74%) were recorded under the treatment of Zn + PBZ over nonsprayed plants. Additionally, Zn and/or PBZ spraying under low and high salinity mitigate the harmful impact of salt stress on vegetative growth. The most effective treatment in mitigation of the injuries of severe salinity was Zn + PBZ that increased SL, SFW, and SDW by 35, 19, and 38% as compared with unsprayed severe salt-affected plants. Data presented in Figure 1a–c revealed that the highest and lowest field pea plant growth was observed in nonsalinized and plants treated by 100 mM NaCl, respectively (Figure 1a–c). The SL, SFW, and SDW were significantly decreased up to 20, 21, and 19% under moderate salinity and by 31, 24, and 23% under severe salinity levels, respectively, relative to nonsalinized plants.

### 2.2. Chlorophyll

The concentration of total chlorophyll was independently influenced (*p* < 0.05) by NaCl or Zn and/or PBZ spray (Figure 1d). The concentration of total chlorophyll was progressively lowered with the increase in NaCl levels corresponding to non-salt-stressed plants that decreased by 48 and 66%, respectively (Figure 1d). Zinc and PBZ spraying not only counteracted the drastic influence of NaCl on chlorophyll concentration, but nevertheless induced a considerable stimulating impact of chlorophyll assimilation compared with those of the corresponding salt-stressed plants. The most effective treatment on enhancing chlorophyll concentration was Zn + PBZ, which increased it by 49% and 114% over the untreated control plants or severe salinity-affected plants, respectively.

### 2.3. Ion Percentage

**Figure 2:**
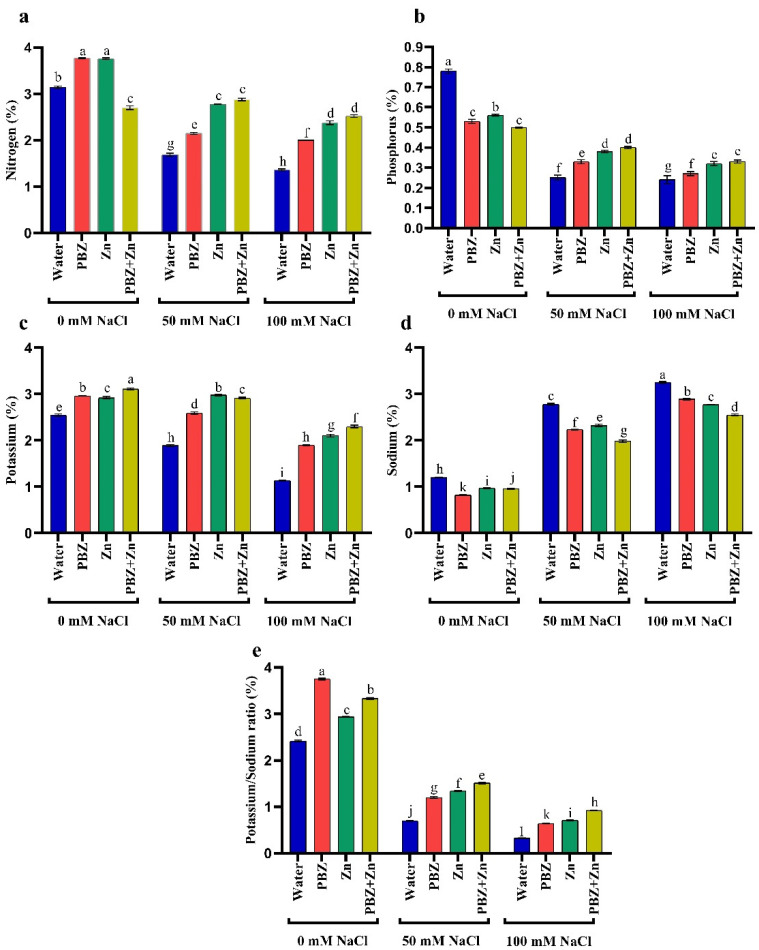
Effect of salinity level (0, 50, 100 mM NaCl) and applications of zinc (Zn), paclobutrazol (PBZ), and their interaction (Zn + PBZ) on (**a**–**d**) ion percentage and (**e**) potassium/sodium ratio of pea plants at 50 days from sowing. Values (*n* = 5) in columns followed by the different letter (a, b, c, d, e, f, g, h, i, j, k, l) are significantly different, *p* < 0.05.

Data presented in Figure 2 indicates that salinity stress decreased NPK and K^+^/Na^+^ in pea shoots. They decreased N by 46 and 57%, P by 66 and 71%, K^+^ by 25 and 55%, K^+^/Na^+^ ratio by 70 and 84% under low and high salinity levels, respectively, compared to nonsalinized treatment. Meanwhile, low and high salinity increased Na^+^ by 150 and 194% over control plants. Alternatively, the application of Zn and/or PBZ considerably increased all ion percentages in pea shoots under the nonsalinized condition (Figure 2a–e). Foliar spray with Zn and/or PBZ under moderate and high salinity levels markedly nullifies their drastic influence on ion percentage. The main effective treatment was Zn+PBZ, which increased shoot N (90%), P (44%), K^+^ (117%), and K^+^/Na^+^ ratio (166%), meanwhile decreasing Na^+^ (24%) relative to nonsprayed severe salt-affected plants.

### 2.4. Antioxidant Solutes

**Figure 3:**
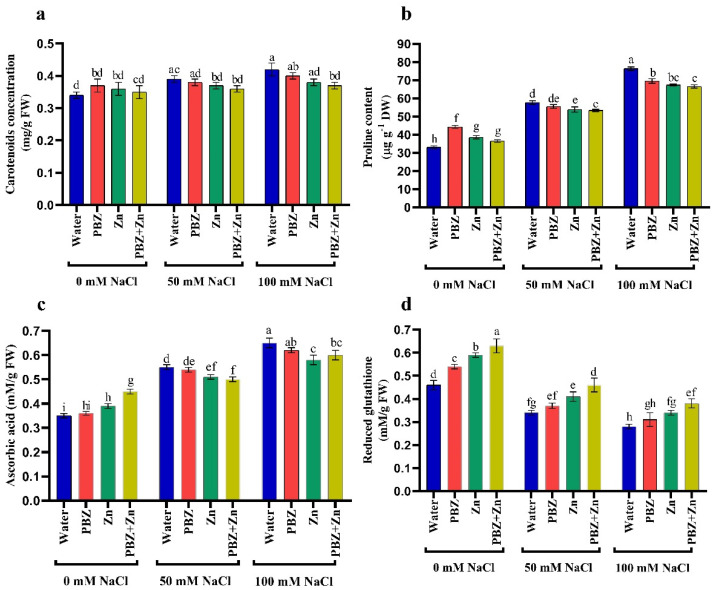
Effect of salinity level (0, 50, 100 mM NaCl) and applications of zinc (Zn), paclobutrazol (PBZ), and their interaction (Zn + PBZ) on (**a**–**d**) antioxidant solutes in pea plant shoots at 50 days from sowing. Values (*n* = 5) in columns followed by the different letter (a, b, c, d, e, f, g, h, i) are significantly different, *p* < 0.05.

To clarify the role of Zn and/or PBZ on a salt-stressed pea, we assessed antioxidant production. Results revealed that salinity considerably raised the concentration of nonenzymatic antioxidants, i.e., CAR, AsA, and PRO concentration, principally under severe salinity over control. Conversely, the foliar spraying with Zn and/or PBZ increased the concentration of CAR, AsA, and PRO under nonstress conditions (Figure 3a–c). Regarding the GSH concentration, Figure 3d illustrates that GSH concentration was decreased by salinity levels; meanwhile, the application of Zn and/or PBZ nullifies the drastic effect of salinity on GSH. Figure 3 also reveals that under nonsalinized conditions, the application of Zn or PBZ significantly increased GSH over untreated control plants.

### 2.5. Activities of Antioxidant Enzymes

**Figure 4:**
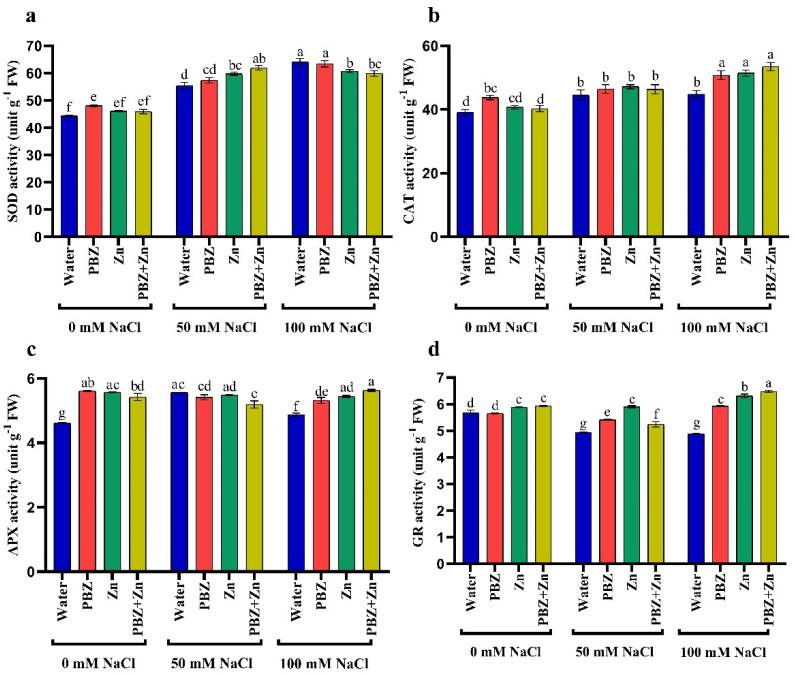
Effect of salinity level (0, 50, 100 mM NaCl) and applications of zinc (Zn), paclobutrazol (PBZ), and their interaction (Zn + PBZ) on (**a**–**d**) antioxidant enzyme activity (unit/g FW/hours) in pea plant shoots at 50 days from sowing. Values (*n* = 5) in columns followed by the different letter (a, b, c, d, e, f, g) are significantly different, *p* < 0.05.

Antioxidant enzyme activity differs significantly under salinity as well as Zn and/or PBZ treatment. Salinity significantly improved superoxide dismutase (SOD), catalase (CAT), and ascorbate peroxidase (APX) activities. High salinity levels had 44%, 14%, and 5% higher SOD, CAT, and APX activity, respectively, than nonsalinized plants (Figure 4a–c). Meanwhile, glutathione reductase (GR) activity was 12% (50 mM NaCl) and 13% (100 mM) lower than nonsalinized plants. Similarly, Zn and/or PBZ application had higher SOD, CAT, APX, and GR activities than nontreated ones under normal conditions. The SOD, CAT, and APX activity was 8%, 11, and 21% higher than nontreated plants, due to the application of Zn; meanwhile, higher GR activity (4%) was obtained under foliar application with Zn + PBZ. The interaction effect of Zn and/or PBZ with salinity on antioxidant enzyme activity showed maximum activity of CAT (36%), APX (22%), and GR (14%) in severe salinity plants treated with Zn + PBZ (Figure 4a–d). However, the maximum SOD (44%) was noted in severe salinity plants without Zn and/or PBZ, compared to the control plants.

### 2.6. Yield Attributes

**Figure 5:**
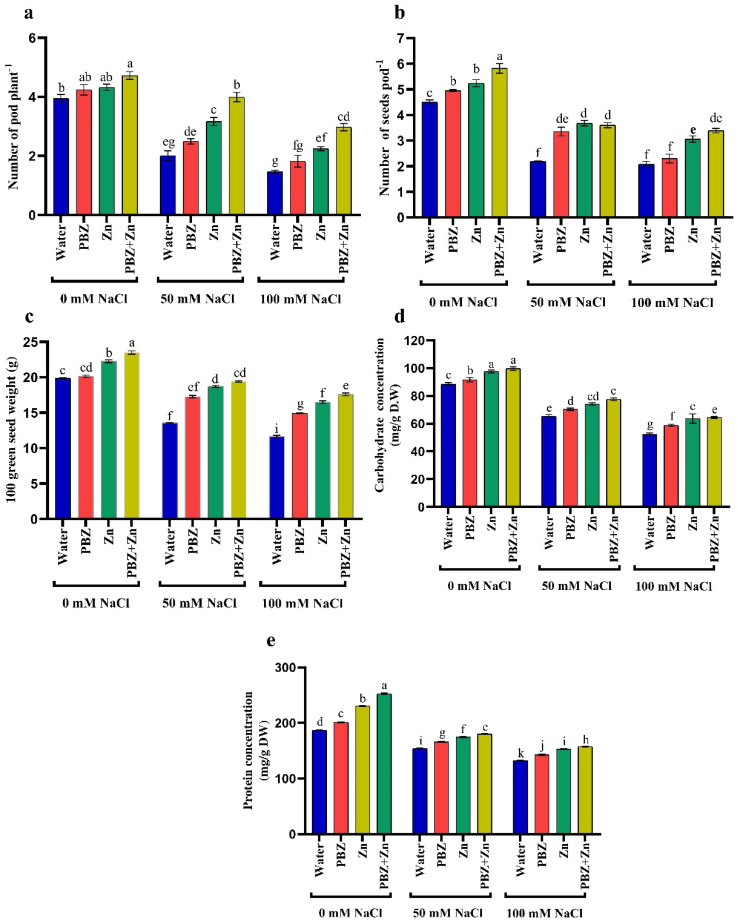
Effect of salinity level (0, 50, 100 mM NaCl) and applications of zinc (Zn), paclobutrazol (PBZ), and their interaction (Zn + PBZ) on (**a**–**c**) pea yield, its quality, (**d**) carbohydrate concentration, (**e**) protein concentration at 75 days from sowing. Values (*n* = 5) in columns followed by the different letter (a, b, c, d, e, f, g, h, i, j, k) are significantly different, *p* < 0.05.

Impact of salinity, Zn, or PBZ on field pea yield, i.e., pod number/plant (PNP), seed number/pod (SNP), 100 green seed weight (GSW), as well as seed carbohydrate and protein concentration, are delineated in Figure 5a–e. It reveals that NaCl levels drastically (*p* < 0.05) lowered pea yield relative to control treatment. Salt stress lowered the PNP by 49% and 62%, SNP by 51% and 53%; 100 GSW by 31% and 41%, carbohydrate concentration in the seeds by 26% and 41%, and protein concentration in the seeds by 17% and 29% under 50 and 100 mM NaCl salinity, respectively, compared with control plants (Figure 5). Conversely, Zn and/or PBZ spray significantly (*p* < 0.05) increased yield attributes proportionate to nonsprayed plants. Zinc and/or PBZ supplementation under salinity mitigated the NaCl injuries on yield components. Under high NaCl levels, Zn + PBZ spraying increased PNP (103%), SNP (62%), 100 GSW (50%), seed carbohydrate concentration (23%), and seed protein concentration (18%) over their respective untreated salt-affected plant (Figure 5a–e).

## 3. Discussion

Plants endure seriously once they are cultivated in saline conditions. The drastic effect of salinity as indicated in the present study on plant growth was confirmed with previous findings for several crops [5,17,25]. The overall plant growth reduction under salinity might result from the destructive effect of NaCl on various physiological pathways and molecular changes, including photosynthesis, nutrient homeostasis, stomatal resistance to water flow, ROS accumulation, changes in the ultrastructure of chloroplast and mitochondria that interfere with normal metabolism, and hormonal imbalance [5,14,26,27]. Earlier research [5,14,26,27] revealed the mitigation effect of either Zn or PBZ on salt injury in different plants. The stimulating effect of Zn under normal or stress conditions is dedicated to accelerating the photosynthesis processes and accelerated photoassimilation translocation into the plant [19,28,29]. Additionally, Zn induces indole acetic acid (IAA) biosynthesis, accelerating cell division and enlargement, and eliminating ROS [14,15,21]. The encouragement role of PBZ on plant growth could be related to its effect on increasing internal carbon dioxide concentration and leaf thickness, enhancing plant cell water retention, and increasing water use efficiency [21].

A remarkable reduction in chlorophyll under NaCl was recorded in several plants [3,4,5]. The decline in chlorophyll under salinity could be attributed to the decrease in chlorophyll biosynthetic or increased enzymatic chlorophyll deprivation [30], as well as the disintegration of the thylakoid membranes and destruction of chlorophyll by different ROS, and changes in chlorophyll protein complexes [4,31]. Additionally, salinity may cause a decline in the concentration of chlorophyll biosynthesis intermediation [32] and decrease the expression of ChlD, Chl H, and Chl I-1 gene encoding subunits of Mg-chelatase [33]. The application of Zn may restore distorted chlorophyll assimilation attributable to sodium chloride (Figure 1d; [3]). Zinc probably keeps chlorophyll assimilation through the protection of the sulphydryl group and improved Mg uptake [34,35,36]. Paclobutrazol application has been documented to boost chlorophyll under normal or stress conditions [21]. This increment may be ascribed to raise the cytokinin concentration [14] that consecutively improved chloroplast differentiation and chlorophyll assimilation, preventing chlorophyll degradation [37]. This may provide a further approach by which Zn or PBZ preserved chlorophyll content under salinity as well as attenuating ROS.

Salinity normally induces ion imbalances that declined NPK and K^+^/Na^+^ ratio, associated with excess accumulation of toxic ions like Na^+^ [5,17]. Regulation of K^+^ uptake and/or avoidance of Na^+^ absorption, efflux of Na^+^, and exploitation for osmotic adjustment is an approach usually possessed by the plant for maintaining a desirable K^+^/Na^+^ ratio that is an important criterion depicting crop salt tolerance. The similarity between K^+^ and Na^+^ resulted in the competitive uptake as the K^+^ transporter lacks discrimination between K^+^ and Na^+^ ions [6,38]. The preservation of ionic homeostasis under salt stress is the requirement to defend the crop against the production of noxious ions, with K^+^ buildup and Na^+^ reaching the lowest concentration in pea plants. Therefore, the control of Na^+^ buildup and consequently an elevated K^+^/Na^+^ may support salt stress tolerance [17]. On the contrary, zinc supplementation improved the ion standing of crops by motivating the translocation and accretion of ions in crop organs [3,28]. Additionally, Zn typically retained ATPase and Na^+^/H^+^antiport, which facilitate Na^+^ compartmentation under saline conditions [28]. Moreover, PBZ normally improves ion content by increasing its uptake from the soil, through improving root activity [39]. The accessible outcome supports the fact that the preservation of elevated K^+^/Na^+^ is necessary to maintain ion homeostasis, which is commonly predictable in salinity tolerance attributes [4]. The elevated shoot K^+^/Na^+^ might have been involved in improving the plant development with Zn and/or PBZ application under the nonsalinized or salinized circumstances. 

Under typical environmental conditions, cells are capable of equilibrating their oxidant and antioxidant capacity. Yet, ROS production under stress can be poisonous and the strict management of ROS is critical to avoid its injuries. Hence, crops have evolved enzymatic and nonenzymatic antioxidants to reduce ROS production. Abundant water- and lipid-soluble antioxidants have an essential and consistent role, performing both nonenzymatic and as substrates in enzyme-catalyzing detoxification reactions. Salinity [4] triggers the accumulation of ROS in different plant species as an important adaptive mechanism; nevertheless, the role of Zn and PBZ on ROS accumulation is still indistinct. The greatest production of ROS under salt stress or activators amendment may be due to altering the ROS biosynthesizing and decomposition enzymes, and/or gene (P5CS1, ProDH) expression, besides accelerating ribulose-1, 5-biphosphate carboxylase (Rubisco), and carbon anhydrase (CA) enzyme activities [4,14,40].

Ascorbic acid (AsA) is a small water-soluble powerful antioxidant in several plants that accumulate about 2–25 mM under normal conditions [41]. Under saline conditions, and Zn [3] and PBZ [21] availability, plants accumulate more AsA in their tissues. Ascorbic acid acts directly to neutralize ROS and contributes to the cellular homeostasis through multiple strategies [41,42]: (1) AsA can eradicate numerous ROS molecules and α-tocopherol, thereby protecting cellular biomembranes; (2) AsA reduces the production of H_2_O_2_ in the stroma; and (3) AsA acts in an imperative role in plant biochemical pathways and developmental processes, including the regulation of cell division and expansion. This diversity of functions has led several studies to distinguish between the role of AsA as a prevailing antioxidant and redox buffer and its role as a signaling molecule occupied in the regulation of complex pathways and their response to stressful conditions [41].

Glutathione (GSH) is a cysteine-containing tripeptide found in nearly all biota. The physiological occupations of glutathione have been principally ascribed to its reduced form. Therefore, the maintenance of an elevated quantity of GSH in plants is a critical requisite [43]. The current research proved that the growth reduction with salinity might be coupled with the overproduction of ROS, as well as a reduction in GSH concentration. On the other hand, the promotive effect of Zn and/or PBZ on plant growth under normal or stressful conditions may be related to the extra production of GSH. These outcomes verify the previous results of Kamran et al. [21] and Sofy et al. [5]. Reduced glutathione was recognized to be related to various developmental stages in plants like cell division, cell differentiation, cell death, and enzymatic upregulation [43]. The defense role of GSHs could be classified into three types [44,45,46]: (1) GSH is a key antioxidant and redox buffer that enhances oxidative stress responses; (2) GSH acts as a substrate of glutathione 3-transferases in detoxification reactions; and (3) GSH involves the restoration of AsA, and ultimately necessary in defensive membranes by preserving α-tocopherol and zeaxanthin in a reduced form.

The positive role of Zn and PBZ on the eradication of ROS may result from the accumulation of carotenoid (CAR) in plant tissues as a common antioxidant. The best recognized antioxidation role of CAR is their capacity to eradicate ROS through physical or chemical elimination [47,48]. Results indicated that PBZ application significantly increased AsA and total glutathione under nonsalinized conditions. Zinc or PBZ application also raised and restored nonenzymatic antioxidants in pea plants compared to the nontreated ones under salinity conditions. It is suggested that the increase in antioxidant contents with the application of Zn or PBZ possibly reduced ROS induced damage, leading to plant adaptation to salinity. The increase in AsA and total glutathione levels in Zn or PBZ treated plants could exhibit the stimulatory effect on the enzymes of the AsA–GSH cycle, especially APX activity, which is important to detoxify H_2_O_2_ overproduction. These contents play an imperative function in controlling the cellular redox state of the antioxidant protection system.

Under stress conditions, the ROS molecules must be detoxified to reduce injury. Proficient devastation of ROS needs the synchronization of numerous antioxidant enzymes [5,7,10,11,12]. The effect of ROS is mitigated by multiple scavenging enzymes, i.e., SOD, CAT, APX, and GR. However, a number of antioxidant enzymes are completely committed to ROS homeostasis, and some others are responsible for the growth, redox regulation of target proteins, and detoxification reactions [4,42]. 

Superoxide dismutase represents the initial line of protection alongside ROS that accelerates the dismutation of O_2_^−^ with large effectiveness, leading to the assembly of H_2_O_2_ and O_2_ [41] that improves the scavenging systems of the cell and declines the buildup of ROS. Commonly, H_2_O_2_ is eradicated by CAT that has an exceedingly elevated turnover number; i.e., each CAT molecule will convert 6 million H_2_O_2_ molecules to H_2_O and O_2_ each second [41,46]. It was reported that the rise in APX activity could result from the activation of pre-existing or assimilation of new APX [49]. The activation of APX and the associated rise in SOD activity advocate that this is an adaptation to eradicate the extra H_2_O_2_ generated. Glutathione reductase (GR) has critical functions in cell defense against ROS, by maintaining the reduced status of GSH and AsA pools that in turn sustain cellular homeostasis under environmental stress [45]. Physiomolecular investigations have revealed that GR is a central enzyme for the eradication of ROS, which is constantly produced in diverse compartments under environmental conditions. Glutathione reductase converts the oxidized glutathione (GSSG) to GSH, maintaining the elevated GSH/GSSG ratio [30]. It could be suggested that the Zn and/or PBZ are superior to GR activity, which possibly will boost NADP^+^/NADPH ratio, and ensures the accessibility of NADP^+^ to receive electrons generated from the flow of electrons to O_2_ and low ROS compound production. Improved GR activity maintain a high GSH/GSSG ratio that is requested for the regulation of AsA threshold rank and the activation of numerous enzymes responsible for CO_2_ fixation [45].

The yearly worldwide monetary loss resulting from salinity is 27.5 billion USD [1]. The decline in crop yield under salt stress was indicated previously by Farouk and Al-Amri [3], and support the results of the current investigation. The reduction in yield under salt stress conditions was possibly caused by the decrease in photoassimilating assembly, and the mobilization of photoassimilates, leading to a reduction in the harvest index [50], pollen viability, and stigmatic receptivity [30]. Nevertheless, the current study showed that Zn or PBZ application improved salinity tolerance and increased pea yield. The role of Zn on crop yield is possibly due to its influence on the improvement of CA activity and Rubisco, which induced CO_2_ assimilation and photosynthetic capacity, leading to maximum dry matter production [29]. Furthermore, Zn has a significant function in sexual reproduction, i.e., the development of floral organs, gametogenesis, and seed formation. Besides, it improves pollen–stigma interaction and pollen tube formation [14]. Mohamed et al. [34] documented that Zn supplementation increased morphological criteria as PNP, SNP, and GSW. Paclobutrazol spray has been observed to boost the yield of various crop species [21]. The positive impact of PBZ on yield possibly results from (1) the increase in canopy size which, in turn, improves light interception and increases photosynthetic rate, and reduces senescence processes [21]; (2) the maintenance of higher rates of photosynthesis with relatively high fluorescence ratio and water use efficiency [21]; and (3) a well-developed root system that determines water and ion uptake and their utilization [5,46]. It has been reported that seed quality was positively affected by Zn [18] or PBZ application [14]. The positive effect of Zn and PBZ on seed carbohydrate content would be related to the increase in starch synthase and CA activity, as well as improved Rubisco activity that improves seed development [51,52,53]. Additionally, zinc application is recognized to preserve enzyme activity through binding the sulphydryl group, and hence defending disulfide formation that leads to a rise in protein biosynthesis and protein content in the seed [34,35,48].

## 4. Materials and Methods

### 4.1. Experimental Design

A randomized complete block design including five replicates was applied at the experimental farm of the Botany Department, Faculty of Science, Al-Azhar University, Cairo, Egypt, throughout the 2017/2018 season, to assess the mitigating effects of zinc sulfate (Zn) and/or PBZ on salt-affected field pea plant productivity and antioxidant strategy. Plastic pots containing 10 kg of air-dried clay loam soil (40% clay, 35% silt, and 25% sand; pH 7.7; electrical conductivity (EC, 1.28 mmhos m^−1^)), supplemented with 2 and 1 g pot^−1^ calcium superphosphate and potassium sulfate, respectively, were prepared and used for the study. Nitrogen fertilizer (ammonium nitrate, 4 g pot^−1^) was added in an equal portion after 20 and 35 days from sowing (DFS). The pots were separated into 3 independent sets (20 pots for each): nonsalinized and salinized with 50 or 100 mM sodium chloride (NaCl). Salinity was induced by adding the appropriate amount of salt to the pot as a water dissolved solution during the first irrigation. Fifteen uncontaminated field pea seeds (*Pisum sativum* L. cv. Master B) were planted in each pot on 10 October. After full emergence (15 DFS), thinning was done to leave five homogenous plants in each pot. Every set was separated into four groups and sprayed with a hand-held sprayer (150 mL plant^−1^) twice (30 and 45 DFS), with water, 100 mg L^−1^ Zn (ZnSO_4_, 7H_2_O), 200 mg L^−1^ PBZ, or Zn + PBZ supplemented with 0.05% Tween 20 as a surfactant.

### 4.2. Measurement of Vegetative Attributes

Shoot length (SL), shoot fresh (SFW) and dry (SDW) weights were recorded at 50 DFS. For measurement of SFW and SDW, the plants were excised and the SFW was assessed directly; then the shoots were dehydrated in an oven (70 °C) for 48 h and reweighted again for SDW.

### 4.3. Physiological and Biochemical Trials

All physiological and biochemical assessment was carried out in plant shoots after 50 DFS. Total chlorophyll and total CAR were assessed in the third upper leaves once extracted with N,N-Dimethyl formamide by a spectrophotometer at 647, 665, and 453 nm, their concentrations were then estimated using the formula of Lichtenthaler [54].

Nitrogen ”N”, phosphorus ”P”, and potassium ”K” were estimated after wet digestion of shoot dry matter [55]. Total N and K were assessed following the micro-Kjeldahl technique and flame photometric method, respectively. The molybdenum-reduced molybdophosphoric blue color technique was followed for P determination. Alternatively, Na^+^ was extracted with boiling water for 3 h and determined flame photometrically [56].

The scheme of Bates, et al. [57] was used to estimate proline (PRO) concentration in fresh tissue and then expressed as µg proline/g FW. The concentration of AsA was estimated according to Deepa, et al. [58] protocol with minor adaptations. Five hundred milligrams of pea shoot tissue was homogenized in 10 mL metaphosphoric acid (MPA, 3% *w*/*v*) at 4 °C for 1 min, and then centrifuged. An aliquot of the supernatant was combined with 5 mL of MPA, after that titrated with 0.1 mM 2,6-dichloroindophenol to the endpoint. The protocol described by Guri [59], using 6 mM 5–5′-dithiobis (2-nitrobenzoicacid) (prepared in 0.1 M K–P buffer with pH 7.5), was followed for the determination of GSH.

For antioxidant enzyme activities, leaf tissue was homogenized in an prechild extraction buffer containing 1 mM ethylene diamine-tetra acetic acid, 1% (*w*/*v*) polyvinylpyrrolidone, 1 mM phenylmethylsulfonyl fluoride, and 0.05% Triton X-100 in 50 mM K-phosphate buffer (pH = 7.0) [60]. Protein concentration was assessed following the Bradford assay procedure [61]. The catalase (CAT, EC 1.11.1.6) and superoxide dismutase (SOD, EC 1.15.1.1) activity were determined as depicted previously [60]. Meanwhile, ascorbate peroxidase (APX, EC 1.11.1.11) activity was determined directly in the fresh extract as indicated by Zhu, et al. [62]. The glutathione reductase (GR, EC 1.6.4.2) activity was assayed following the Foyer and Halliwell [63] method with minor modifications.

At harvesting (75 DFS), pod number per plant (PNP), seed number per pod (SNP), and 100 green seed weight (GSW) were estimated; moreover, a well-dried pea seed powder was used for the assessment of protein and carbohydrate concentrations [64].

### 4.4. Statistical Analysis

Data were introduced as the mean of five replicates ± standard error. Statistical analysis was performed using Costat software (CoHortSoftware, 2006; Monterey, CA, USA), and the means were compared with Duncan’s multiple range tests. Letters on top of the columns in the figures indicate the significant difference at the *p* ≤ 0.05 levels among treatments.

## 5. Conclusions

Spraying of Zn and PBZ increased chlorophyll concentration, improved antioxidant capacity, decreased sodium buildup, and prohibited NaCl-induced K^+^ leakage, thus preserving a superior K^+^/Na^+^ ratio that ultimately enhanced plant growth and productivity. In conclusion, spraying salt-affected pea plants with 100 mg L^−1^ zinc sulfate plus 200 mg L^−1^ paclobutrazol twice at 30 and 45 days from sowing could be a hopeful method for counteracting the injuries of salinity by activating the antioxidant defense system, and thus improving crop yield and its quality.
